# Long-stay pediatric patients in Japanese intensive care units: their significant presence and a newly developed, simple predictive score

**DOI:** 10.1186/s40560-019-0392-2

**Published:** 2019-07-29

**Authors:** Emily Knaup, Nobuyuki Nosaka, Takashi Yorifuji, Kohei Tsukahara, Hiromichi Naito, Hirokazu Tsukahara, Atsunori Nakao, for the JaRPAC Study Group

**Affiliations:** 1 Department of Emergency, Critical Care and Disaster Medicine, Graduate School of Medicine, Dentistry and Pharmaceutical Sciences, Okayama University, Okayama, Japan; 2 Department of Pediatrics, Graduate School of Medicine, Dentistry and Pharmaceutical Sciences, Okayama University, Okayama, Japan; 3 Department of Pediatrics, Cedars-Sinai Medical Center, Los Angeles, USA; 4 Department of Human Ecology, Graduate School of Environmental and Life Science, Okayama University, Okayama, Japan

**Keywords:** Decision support, Intensive care, Length of stay, Mortality, Outcome, Pediatric, Risk, Prediction rules, Scoring system

## Abstract

**Background:**

The length of stay (LOS) in intensive care units (ICUs) has been used as a good indicator not only for resource consumption but also for health outcomes of patients. However, data regarding pediatric LOS in Japanese ICUs are limited. The primary aim of this study was to characterize the Japanese pediatric ICU patients based on their LOS. Second, we aimed to develop a simple scoring system to predict long-stay pediatric ICU patients on admission.

**Methods:**

We performed a retrospective cohort study using consecutive pediatric data (aged < 16 years) registered in the Japanese Registry of Pediatric Acute Care (JaRPAC) from October 2013 to September 2016, which consisted of descriptive and diagnostic information. The factors for long-stay patients (LSPs; LOS > 14 days) were identified using multiple regression analysis, and subsequently, a simple predictive scoring system was developed based on the results. The validity of the score was prospectively tested using data from the JaRPAC registration from October 2016 to September 2017.

**Results:**

Overall, 4107 patients were included. Although LSPs were few (8.0% [*n* = 330]), they consumed 38.0% of ICU bed days (9750 for LSPs versus 25,659 overall). Mortality was seven times higher in LSPs than in short-stay patients (9.1% versus 1.3%). An 11-variable simple predictive scoring system was constructed, including Pediatric Index of Mortality 2 ≥ 1 (2 points), liver dysfunction (non-post operation) (2 points), post-cardiopulmonary resuscitation (1 point), circulatory disorder (1 point), post-operative management of liver transplantation (1 point), encephalitis/encephalopathy (1 point), myocarditis/cardiomyopathy (1 point), congenital heart disease (non-post operation) (1 point), lung tissue disease (1 point), Pediatric Cerebral Performance Category scores ≥ 2 (1 point), and age < 2 years (1 point). A score of ≥ 3 points yielded an area under the receiver operating characteristic curve (AUC) of 0.79, sensitivity of 87.0%, and specificity of 59.4% in the original dataset. Reproducibility was confirmed with the internal validation dataset (AUC 0.80, sensitivity 92.6%, and specificity 60.2%).

**Conclusions:**

Pediatric LSPs possess a significant presence in Japanese ICUs with high rates of bed utilization and mortality. The newly developed predictive scoring system may identify pediatric LSPs on admission.

**Electronic supplementary material:**

The online version of this article (10.1186/s40560-019-0392-2) contains supplementary material, which is available to authorized users.

## Background

Long-stay patients (LSPs) in intensive care units (ICUs) are generally accepted as being susceptible to higher mortality than short-stay patients (SSPs) [1]. In addition, they consume a higher amount of medical costs, resources, and ICU capacity [2]. Since the 1980s, when pediatric LSPs in ICUs first got attention in research [3], the number of pediatric LSPs in ICUs has been increasing, with the development of life-supporting skills and technologies such as the availability of the pediatric advanced life support course or extracorporeal membrane oxygenation therapy [4]. Multiple studies reported that they are responsible for more than one third of ICU bed days although the proportion of LSPs among the entire admitted population is smaller than 10% [3, 5–7]. It is often the case for pediatric LSPs that their functional outcomes are unfavorable (moderate or severe disabilities, and death) [8]. Although these basic data are essential for discussion to improve the current ICU practices for pediatric patients, Japan has little data about pediatric LSPs in the ICU.

Predicting LSPs in ICUs on admission has been another interest of research for decades [6, 9–12]. Appropriate prediction might provide practical assistance for proactive patient management in ICUs [11]. Health care teams could be dedicated to communicate and purvey sufficient information to families and clinicians could deliberately provide preventive care against different complications. ICU staff could systematically schedule bed use and medical resources. Experienced ICU staff might be able to predict pediatric LSPs on admission; however, this would be unreliable. In fact, clinicians could predict LSPs less correctly than SSPs in adult ICU settings [13]. There have been several studies in the USA that attempted to develop predictive models for the length of stay (LOS) of pediatric patients in the ICU [5, 6]. However, it is easily assumed that these foreign models have limited applicability to pediatric populations in Japanese ICUs because of the wide differences in common diseases among children and health care systems [14, 15]. Moreover, the discussion regarding LSP prediction for pediatric patients in the ICU is still poor in Japanese ICUs.

The paucity of data prompted us to survey the pediatric LSP population in Japanese ICUs and to create a useful system to identify them in the early ICU course, using consecutive pediatric data (< 16 years old) registered in the Japanese Registry of Pediatric Acute Care (JaRPAC).

## Methods

### JaRPAC overview

JaRPAC is an observational, multicenter, prospective database that contains data on consecutive pediatric patients admitted to ICUs in Japan. The registry was started on October 1, 2013. The study is mainly led by the pediatric committee of the Japanese Society for Emergency Medicine and the study website can be reached at jarpac.org. The JaRPAC registry includes information on admitted patients to ICU or those who died in the emergency room. The subjects in children’s hospitals are all registered regardless of their age, while those in other hospitals are registered if their age is less than 16 years. Otherwise, no exclusion criteria are applied to the registry. Using application software designated for this database, patients’ data are collected with 23 variables at admission and 27 variables at discharge from ICU, including data regarding pre-hospital management, the predictive death rate, demographics, treatments, and outcomes. The software equips automated logic checks to avoid miss-hit or data deficiencies. The registry is securely held at the National Center of Child Health and Development (NCCHD) and anonymized data are provided to the principal investigator on request with a specific study protocol that was approved by the JaRPAC steering committee. The study results are available to the public in the form of annual reports, conference abstracts, and peer-reviewed publications.

### Patient data

We obtained the following 3 year JaRPAC data of consecutive patients aged less than 16 years who were admitted to ICU from October 2013 to September 2016 to survey the characteristics of pediatric LSPs in ICUs (analysis dataset): sex, age in month, dates of admission and discharge from the ICU, reasons for ICU admission, diagnosis at discharge, disease category, elective/emergency admission status, cardiopulmonary resuscitation (CPR) before admission or during the ICU stay, Pediatric Index of Mortality 2 (PIM2) [16], chromosome abnormalities, discharge disposition, and Pediatric Cerebral Performance Category (PCPC) score [17] before admission and at discharge from the ICU. The Injury Severity Score (ISS) [18] was extracted for all patients with injuries. JaRPAC codes the reason for admission into seven categories: postoperative management, post CPR, respiratory distress, circulatory disorder, neurological disorders, monitoring/assessment, and others. Allowable values for discharge disposition in JaRPAC are defined within five categories: general ward, back transferred to the original hospital, transferred to another hospital, home, and expired. Furthermore, JaRPAC categorizes diseases in a three-step manner; an additional table file shows this in more details (see Additional file 1). PIM2 and PCPC scores are the fundamental tools used worldwide for the evaluation of pediatric patients in the ICUs. Briefly, PIM2, the calculation system for predicting mortality rates for pediatric patients, uses 10 variables. The PCPC score is a tool for quantifying short-term cognitive impairments in pediatric patients; it has a scale ranging from 1 to 6 (1 = normal, 2 = mild disability, 3 = moderate disability, 4 = severe disability, 5 = coma or vegetative state, and 6 = brain death).

It should be noted that although the JaRPAC registry includes information on patients who died in the emergency room, we excluded the data of these patients at the point of data extraction. Furthermore, we did not exclude any other patients while creating the prediction model for LSPs. This means that our analysis included data of patients who died within several days after ICU admission in SSPs because the clinicians could not predict whether patients would die at the point of their ICU admission.

In total, seven facilities registered their patients’ data in the JaRPAC database during our study period. The characteristic breakdown of these facilities is as follows: (1) four pediatric ICUs (4–20 beds; a total of 38 beds) and (2) three general (mixed adult-pediatric) ICUs (12–20 beds; a total of 52 beds). There were no ICUs classified as “cardiac ICUs.”

We obtained the same JaRPAC data as described above from the same facilities included in the original dataset for consecutive patients (< 16 years) from October 2016 to September 2017 to test the accuracy of prediction for the newly developed score as we shall explain later; these data represented the “internal” validation dataset. In addition, we obtained the same data from facilities newly joined during this period (not included in the original dataset, one general PICU [10 beds] and one general adult-mixed ICU [8 beds]); these data represented the “external” validation dataset.

### Defining the LSPs

LOS was calculated by subtracting the date of ICU admission from the date of ICU discharge plus one. The definition of LSP was determined considering various indexes by following the techniques described in similar studies [19, 20]: (1) beyond five times the median LOS, (2) beyond the 95th percentile of the LOS, (3) greater than two standard deviations from the mean in Gaussian distribution, and (4) beyond the visually recognized threshold from the “tail” portion of the LOS histogram in the case of skewed distribution.

### Statistical analysis

After we examined the distribution of LOS in ICUs to define the LSP, we conducted a descriptive analysis of the participants overall or stratified by the LSP status. We then compared the risk of mortality (i.e., expired) obtained from the discharge disposition as clinical outcomes between LSPs and SSPs. We also calculated the rate of mortality considering the patient-day as a denominator, stratified by the LOS.

We then created a prediction model for LSPs. We conducted a backward selection stepwise logistic regression model and used the significance level of *p* = 0.05 for removal of variables from the model. When a least-significant variable was non-significant, the variable was removed and the model was re-estimated; the final model was developed when all of the variables were significant. We entered the following variables in the model in the beginning: age (dichotomous, < 2 years or ≥ 2 years); emergency admission (dichotomous); post CPR admission (dichotomous); admission due to circulatory disorders (dichotomous); disease category of myocarditis, congenital heart disease, encephalopathy/encephalitis, liver dysfunction, lung tissue disorders, or multiple trauma; PIM2 (dichotomous, < 1 or ≥ 1); PCPC score on admission (dichotomous, < 2 or ≥ 2); or ISS (dichotomous, < 10 or ≥ 10). We selected the two reasons for admission and six disease categories because they predicted the LSPs based on the descriptive analysis. We then removed sex and chromosome abnormalities because the validation dataset did not include these variables. After selecting variables using a logistic regression model, we estimated odds ratios (ORs) with 95% confidence intervals (CIs) for each of the remaining variables and computed the area under the receiver operating characteristic (ROC) curve (AUC). To increase the usefulness of the prediction model, we assigned integer score points to each remaining variable based on the ORs and created a prediction rule for the LSPs, that assigned the defined integer score points based on the presence of each variable for the patients. We calculated the sum of all points for each patient in the current dataset, as well as, the validation dataset and again calculated the AUC. We also calculated the sensitivity and specificity of the prediction rule after defining the certain cutoff value based on the ROC curve.

Stata SE version 15 (StataCorp, College Station, TX, USA) was used for all analyses. This study was approved by the Okayama University Graduate School of Medicine, Dentistry and Pharmaceutical Sciences Institutional Review Board (Eki853 and Ken1812-027).

## Results

There were 4108 ICU admissions during the 3 year study period included in the analysis dataset. Of these admissions, just one case was excluded because the dates of admission and discharge were obviously incorrect. Thereafter, the data for 4107 patients were used as the final analysis dataset.

### LSP definition

**Fig. 1:**
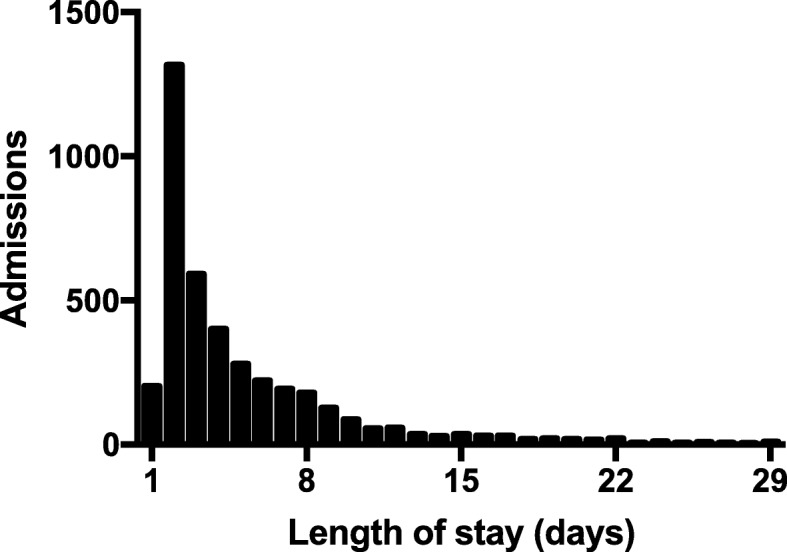
Distribution of pediatric length of stay in intensive care units. Forty data points (91 patients) are outside the *x*-axis limits

Figure 1 portrays the frequency distribution of patients based on the length of stay in the ICU. The median and mean LOS were 3.0 (minimum to maximum, 1–370) and 6.2 ± 11.7 days, respectively. Considering its rightward skewed distribution, we calculated or visually identified three indexes described above to decide upon the clinical threshold for prolonged ICU stays: (1) 15 days for five times the median LOS, (2) 19 days as the 95th percentile of LOS, and (3) 13 days as the start of the “tail” of the distribution shown in Fig 1. Taken together these results, for the purpose of practical convenience, we defined LSPs as patients who had a LOS in the ICU greater than 14 days.

### Characteristics of the study population on admission

Table 1 shows the patients’ characteristics on admission. Overall, 42.9% (*n* = 1762) of patients were less than 2 years old and the majority of patients had emergency admissions (55.4%, *n* = 2277). Postoperative management was the most common reason for the ICU admission (44.2%, *n* = 1814). Most patients had an endogenous disease (91.6%, *n* = 3760) and had a good functional status before ICU admission (76.4%, *n* = 3136, PCPC 1 and 2). Chromosomal abnormality was reported among 6.4% (*n* = 263) of patients, 59.7% (*n* = 157) of whom were 21 trisomy.

**Table 1:** Characteristics of pediatric patients on admission in Japanese intensive care units

		All patients *n* = 4107		SSPs *n* = 3777		LSPs *n* = 330		Sex- and age-adjusted OR of LSPs compared to SSPs (95% CI)		Sex- and age-adjusted regression coefficient (95% CI)	
Age (month), median (IQR)		23.0 (7.0–71.0)		24.0 (8.0–71.5)		10.0 (3.0–53.3)					
Male [numbers (%)]		2362	(57.5%)	2165	(57.3%)	197	(59.7%)				
Emergency admission [numbers (%)]		2277	(55.4%)	2042	(54.1%)	235	(71.2%)	2.38	(1.83–3.08)*	2.39	(1.66–3.11)*
Reasons for admission [numbers (%)]											
Post-operative		1814	(44.2%)	1730	(45.8%)	84	(25.5%)	1	(Reference)		(Reference)
Post-CPR		84	(2.0%)	59	(1.6%)	25	(7.6%)	9.29	(5.51–15.65)	7.53	(5.00–10.07)
Respiratory disorders		833	(20.3%)	763	(20.2%)	70	(21.2%)	1.88	(1.35–2.61)	1.82	(0.87–2.77)
Circulatory disorders		243	(5.9%)	193	(5.1%)	50	(15.2%)	5.23	(3.57–7.67)	4.12	(2.57–5.67)
Neurological disorders		626	(15.2%)	578	(15.3%)	48	(14.5%)	1.80	(1.25–2.61)	1.86	(0.80–2.91)
Others		507	(12.3%)	454	(12.0%)	53	(16.1%)	2.58	(1.80–3.70)	2.06	(0.92–3.20)
Diagnosis at admission [numbers (%)]											
CHD (all)		523	(12.7%)	469	(12.4%)	54	(16.4%)	1.22	(0.89–1.67)**	1.75	(0.66–2.84)**
Post-operation		470	(11.4%)	431	(11.4%)	39	(11.8%)	0.92	(0.65–1.32)**	1.26	(0.12–2.40)**
Non-post operation		53	(1.3%)	38	(1.0%)	15	(4.5%)	3.92	(2.12–7.25)**	5.02	(1.86–8.20)**
Myocarditis/cardiomyopathy		52	(1.3%)	34	(0.9%)	18	(5.5%)	6.16	(3.43–11.08)**	6.85	(3.67–10.03)**
Encephalitis/encephalopathy		135	(3.3%)	110	(2.9%)	25	(7.6%)	2.84	(1.81–4.47)**	7.98	(6.00–9.97)**
Liver dysfunction (all)		212	(5.2%)	155	(4.1%)	57	(17.3%)	4.72	(3.39–6.56)**	6.28	(4.68–7.88)**
Post-transplantation		97	(2.4%)	72	(1.9%)	25	(7.6%)	4.07	(2.54–6.53)**	7.45	(5.11–9.79)**
Post-status of the other operation		29	(0.7%)	29	(0.8%)	0	(0%)	1.00		− 3.28	(− 7.01 to 0.44)**
Non post-operation		77	(1.9%)	45	(1.2%)	32	(9.7%)	8.63	(5.39–13.84)**	8.98	(6.36–11.59)**
Upper airway obstruction		198	(4.8%)	184	(4.9%)	14	(4.2%)	0.86	(0.49–1.50)**	− 1.32	(−2.98 to 0.35)**
Lower airway obstruction		236	(5.7%)	230	(6.1%)	6	(1.8%)	0.28	(0.12–0.63)**	− 0.29	(− 1.82 to 1.25)**
Lung tissue disease		241	(5.9%)	211	(5.6%)	30	(9.1%)	1.74	(1.16–2.61)**	1.89	(0.38–3.41)**
Disordered control of breathing		48	(1.2%)	44	(1.2%)	4	(1.2%)	0.96	(0.34–2.70)**	0.01	(− 3.31 to 3.33)**
Trauma		226	(5.5%)	218	(5.8%)	8	(2.4%)	0.45	(0.22–0.92)**	− 1.91	(− 3.49 to − 0.35)**
Head injury		132	(3.2%)	126	(3.3%)	6	(1.8%)	0.54	(0.23–1.23)**	− 1.97	(− 3.99 to 0.05)**
Multiple injury		25	(0.6%)	23	(0.6%)	2	(0.6%)	1.20	(0.28–5.14)**	− 0.18	(− 4.78 to 4.42)**
ISS, median (IQR)		10 (4–17)		10 (4–17)		17 (10–40)		1.04	(1.01–1.07)	0.19	(0.16–0.22)
								Per 1.0 increase		Per 1.0 increase	
PIM2, median (IQR)		1.0 (0.3–3.0)		0.9 (0.2–2.2)		5.3 (2.1–19.5)		1.04	(1.04–1.05)	0.17	(0.13–0.21)
								Per 1.0 increase		Per 1.0 increase	
PCPC before admission											
[Numbers (%)]	1	2733	(66.5%)	2515	(66.6%)	218	(66.1%)	1	(Reference)		(Reference)
	2	403	(9.8%)	367	(9.7%)	36	(10.9%)	1.17	(0.81–1.70)	− 0.33	(− 1.55 to 0.89)
	3	423	(10.3%)	392	(10.4%)	31	(9.4%)	0.91	(0.62–1.35)	− 0.4	(− 1.59 to 0.80)
	4	537	(13.1%)	494	(13.1%)	43	(13.0%)	1.12	(0.79–1.59)	− 0.3	(− 1.38 to 0.79)
	5	11	(0.3%)	9	(0.2%)	2	(0.6%)	2.95	(0.63–13.84)	13.43	(6.54–20.33)
Chromosomal abnormality											
[Numbers (%)]	None								(Reference)		(Reference)
	21 trisomy	157	(3.8%)	147	(3.9%)	10	(3.0%)	0.72	(0.37–1.39)	− 0.67	(− 2.55 to 1.20)
Others		106	(2.6%)	92	(2.4%)	14	(4.2%)	1.77	(0.99–3.15)	1.00	(− 1.27 to 3.26)

*CHD* congenital heart disease, *CI* confidence interval, *CPR* cardio-pulmonary resuscitation, *IQR* interquartile range, *ISS* Injury Severity Score, *LSPs* long-stay patients, *NE* not examined, *OR* odd ratio, *PCPC* Pediatric Cerebral Performance Category, *PIM2* Pediatric Index of Mortality 2, *SSPs* short-stay patients

*The reference is the planned admission

**The reference is the other diagnoses

LSPs accounted for 8.0% (*n* = 330) of all patients. The proportion of LSP patients who were less than 2-years old was even greater than that of SSPs (60.9% versus 41.3%). The admission reasons of post-CPR and circulatory disorders were more common in LSPs. In terms of diagnosis, those with liver dysfunction, myocarditis/cardiomyopathy, CHD, encephalitis/encephalopathy, and lung tissue disease (pneumonia) were more likely to be LSPs. There was no correlation between PCPC before admission and LOS.

### Clinical outcomes

Table 2 summarizes the clinical outcomes of the study population. LSPs consumed 38.0% of the ICU bed days. Mortality was seven times higher in LSPs than SSPs. Additionally, mortality during the first 0–14 ICU admission days was 0.0024/patient-days, while mortality after 14 ICU admission days was 0.0058/patient-days. Thus, if a patient stayed in the ICU for more than 14 days, mortality per day would increase by approximately 2.5 times that of a patient who stayed in the ICU for 14 days or less. After discharge from the ICU, most patients were managed in the same hospital as the ICU.

**Table 2:** Clinical outcomes of pediatric patients in intensive care units in Japan

	All Patients *n* = 4107		SSPs *n* = 3777		LSPs *n* = 330	
Length of stay (days)						
Average (SD)	6.2 (11.7)		4.2 (2.9)		29.6 (31.8)	
Median (IQR)	3.0 (2.0–7.0)		3.0 (2.0–6.0)		21.0 (17.0–31.0)	
ICU bed days [numbers (% total)]	25659		15909 (62.0%)		9750 (38.0%)	
Mortality [numbers (%)]	79	(1.9%)	49	(1.3%)	30	(9.1%)
Discharge route [numbers (%)]						
General ward	3649	(88.8%)	3390	(89.8%)	259	(78.5%)
Transfer to original hospital	116	(2.8%)	90	(2.4%)	26	(7.9%)
Transfer to new hospital	51	(1.2%)	40	(1.1%)	11	(3.3%)
Home	212	(5.2%)	208	(5.5%)	4	(1.2%)
PCPC on discharge [numbers (%)]						
1	2572	(62.6%)	2418	(64.0%)	154	(46.7%)
2	426	(10.4%)	395	(10.5%)	31	(9.4%)
3	435	(10.6%)	402	(10.6%)	33	(10.0%)
4	557	(13.6%)	498	(13.2%)	59	(17.9%)
5	32	(0.8%)	14	(0.4%)	18	(5.5%)
6	85	(2.1%)	50	(1.3%)	35	(10.6%)
Delta PCPC* [numbers (%)]						
0	3871	(94.3%)	3633	(96.2%)	238	(72.1%)
1	66	(1.6%)	52	(1.4%)	14	(4.2%)
2	48	(1.2%)	29	(0.8%)	19	(5.8%)
3	38	(0.9%)	17	(0.5%)	21	(6.4%)
4	28	(0.7%)	9	(0.2%)	19	(5.8%)
5	48	(1.2%)	29	(0.8%)	19	(5.8%)
Unknown	8	(0.2%)	8	(0.2%)	0	(0)

*PCPC on discharge – PCPC before admission

*IQR* interquartile range, *LSPs* long-stay patients, *PCPC* Pediatric Cerebral Performance Category, *SD* standard deviation, *SSPs* short-stay patients

### Predictive model for LSPs on admission

In the first stepwise logistic regression model, all the remaining variables had ORs more than one as expected from the descriptive analysis, so we accepted the model. The AUC was 0.82 and we showed the remaining variables in Table 3. As liver dysfunction (non-post operation) and PIM2 (≥ 1) had ORs of more than 5, we assigned a score of 2 to the variables and a score of 1 to the other variables. Thus, we ascertained the following prediction rule for LSPs.

$$ \mathrm{Predictivescore}=\mathrm{PIM}2\left(\ge 1\right)\times 2+\mathrm{liverdysfunction}\left(\mathrm{non}-\mathrm{postoperation}\right)\times 2+\mathrm{postCPR}\times 1+\mathrm{circulatorydisorder}\times 1+\mathrm{liverdysfunction}\left(\mathrm{post}-\mathrm{operativemanagementoflivertransplantation}\right)\times 1+\mathrm{encephalitis}/\mathrm{encephalopathy}\times 1+\mathrm{myocarditis}/\mathrm{cardiomyopathy}\times 1+\mathrm{CHD}\left(\mathrm{non}-\mathrm{postoperation}\right)\times 1+\mathrm{lungtissuedisease}\times 1+\mathrm{PCPCbeforeadmission}\left(\ge 2\right)\times 1+\mathrm{age}\left(<2\mathrm{years}\right)\times 1 $$

**Table 3:** Predictive Score for pediatric long-stay patients (score ≥ 3)

	Odds ratio	(95% CI)	Score
Reasons for admission			
Post CPR	4.60	(2.75–7.68)	1
Circulatory disorder	2.11	(1.34–3.31)	1
Disease category			
Liver dysfunction (non-post operation)	9.24	(5.55–15.38)	2
Post-operative management of liver transplantation	4.31	(2.58–7.20)	1
Myocarditis/cardiomyopathy	3.15	(1.57–6.34)	1
Encephalitis/encephalopathy	3.16	(1.95–5.13)	1
CHD (non-post operation)	2.44	(1.18–5.04)	1
Lung tissue disease (pneumonia)	1.80	(1.15–2.80)	1
Other factors			
PIM2 ≥ 1	10.97	(6.62–18.19)	2
Age < 2 years	1.60	(1.23–2.08)	1
PCPC ≥2 before admission	1.36	(1.03–1.79)	1

*CHD* congenital heart disease, *CI* confidence interval, *CPR* cardio-pulmonary resuscitation, *PCPC* Pediatric Cerebral Performance Category, *PIM2* Pediatric Index of Mortality 2

After we applied the prediction rule to the patients in the current dataset, the AUC was 0.79. When we used the cutoff point of 3 based on the ROC curve, the sensitivity and specificity for the LSPs were 87.0% and 59.4%, respectively. When we applied the prediction rule to the “internal” validation dataset (*n* = 378), the AUC was 0.80 and the sensitivity and specificity of LSPs were 92.6% and 60.2%, respectively, using the same cutoff point. Furthermore, when we applied the prediction rule to the “external” validation dataset (*n* = 295), the AUC was 0.76 and the sensitivity and specificity of LSPs were 91.7% and 53.9%, respectively, using the same cutoff point.

As the OR of PIM2 (≥ 1) was relatively higher than that of any other variables, we examined the predictive ability of PIM2 by itself for LSPs. PIM2 and patients’ LOS had a negligible correlation (*r* = 0.20, Additional file 2). Moreover, the AUC was 0.71 when we applied PIM2 as a prediction rule for LSPs; this was lower than that of our newly developed prediction rule.

## Discussion

In this study, we have newly provided an overview of pediatric long-stay ICU patients in Japan using the JaRPAC, a nationwide and multicenter database. Additionally, we have provided a unique and simple predictive scoring system using available data at the point of ICU admission to prospectively identify pediatric LSP-candidates. We showed that pediatric LSPs have a significant presence in Japanese ICUs with high rates of bed utilization and mortality. Eight percent of the entire population used almost 40% of the bed days. Additionally, the mortality in pediatric LSPs was significantly higher. These data signify the need for further interventions for improved LSP outcomes. Thus, identifying LSP candidates on ICU admission will help clinicians to intervene with such patients in the early course of the ICU stay. Although there have been multiple studies which provided prediction models for pediatric LSPs, our newly developed prediction system is so practical that clinicians could identify pediatric LSP candidates by simply adding the scores assigned to the risk factors.

Table 4 provides a summary of previous studies conducted regarding pediatric LSPs in ICUs; a study with a specific condition (post-cardiac surgery) was excluded. All studies were based on pediatric ICUs, and most of them were single-center studies. Even though the definition of LSPs differed from study to study, in all the studies, LSPs were commonly described as occupying excessive ICU bed days (18.5–63%) despite their smaller population frequencies (1.0–7.3%). Moreover, the mortality among LSPs was higher than that among SSPs in all studies. Comparable results were obtained from our study, although in this study, data from multiple mixed adult-pediatric ICUs were included. The differences in outcomes between pediatric ICUs and mixed adult-pediatric ICUs should be investigated in further studies.

**Table 4:** Summary of the published studies on pediatric long-stay patients

Study	Author, year	Country	Study period	No. of ICUs	ICU characteristics	Study population	Exclusion criteria
1	Pollack et al., 1987 [3]	USA	1981–2, 1984–5	1	Multidisciplinary PICUs	647	NA
2	Marcin et al., 2001 [6]	USA	NA	32	General PICUs	11,165	NA
3	van der Heide et al., 2004 [10]	Netherlands	1992–1997	1	Multidisciplinary PICUs	34	Age < 1 year old, >18 years old
4	Naghib et al., 2010 [7]	Netherlands	2003–2005	1	Multidisciplinary PICUs	2607	None
5	Namachivayam et al., 2012 [8]	Australia	1989–2008	1	Tertiary PICUs	27,536	NA
6	Nupen et al., 2017 [17]	South Africa	NA	1	Multidisciplinary PICUs	1126	NA
7	Pollack et al., 2018 [5]	USA	2011–2013	8	General and cardiac PICUs	10,078	Age > 18 years old
8	Knaup et al., 2019	Japan	2013–2016	7	General PICUs and mixed adult-pediatric ICUs	4107	Age > 16 years old
Study	LSP definition	LSP number	LSP age		LOS of LSPs (days)	Bed occupancy by LSPs	Mortality (SSPs/LSPs)
1	> 13 days	7.1% (*n* = 46)	13 months*		35.8**	50.0%	7.3%/17.4%
2	> 12 days	4.7% (*n* = 460)	45.5 months^*^		28.3*	36.1%	4.4%/15.0%
3	≥ 30 days	NA (*n* = 19)	10.7 years**		37**	NA	NA/10.5%
4	≥ 28 days	4.4% (*n* = 116)	1 month**		56*	63.0%	4.6%/22.0%
5	≥ 28 days	1.0% (*n* = 269)	4.2 months^*^		40*	18.5%	NA
6	> 19 days	4.8% (*n* = 54)	4.0 months*		29.5*	30.4%	2.4%/29.6%
7	≥ 19 days	4.6%	NA		NA	37.6%	NA
8	> 14 days	8.0% (*n* = 330)	10 months*		21*	38.0%	1.3%/9.1%

*Median

**Mean

*ICU* intensive care unit, *LOS* length of stay, *LSP*(*s*) long-stay patient(s), *NA* not available, *SSPs* short-stay patients, *USA* the United States of America

Table 5 summarizes the predictors of LSP or independent LSP-associated factors based on the findings of previous studies. Our study and previous studies include similar LSP predictors or LSP-associated factors including pneumonia [6] (i.e., lung tissue disease) and the existence of underlying diseases [5, 6] (e.g., chronic total parenteral nutrition, tracheostomy, intracranial catheter, and higher PCPC). Intriguingly, our study showed post-CPR status as one of the LSP predictors, whereas no CPR-need prior to admission was identified as an LSP predictor in an American study [6]. This might have been caused by the relatively low acceptance of treatment withdrawal in Japan. The mortality in pediatric ICUs after out-of-hospital cardiac arrest (OHCA) was reported to be as high as 45–50% in Western countries [21, 22], whereas this was as low as 34.1% based on our data, as shown in Additional file 3. Conversely, remarkably high rates of poor neurological outcomes at ICU discharge (PCPC = 4 or 5) in LSPs were seen in our analysis (Additional file 3). A study from a single center in the USA showed that 81% of the modes of death after pediatric OHCA were classified as brain death or withdrawal due to neurological prognosis after a median ICU admission of 4 days [22]. Given the fact that the guidelines for treatment withdrawal have only recently been published in Japan [23], it is a stretch to assume that the choice to withdraw from treatment for pediatric patients has been widely accepted in daily ICU management in Japan. However, further validations are required to corroborate this hypothesis.

**Table 5:** Predictors of long-stay pediatric patients or independent long-stay patient-associated factors

Study*	Factors	
2 [6]	• Age < 12 months • Previous ICU admission • Emergency admission • No CPR before admission • Pneumonia • Chronic TPN • Chronic tracheostomy	• Admission from another ICU • Other respiratory diseases • Never discharged from the hospital • Ventilator • Intracranial catheter • 10 < PRISM III—24 score < 33
6 [17]	• Female gender	
7 [5]	• Age < 12 months • Moderate or severe baseline FFS • PRISM III total score • ECMO during PICU stay • RRT during PICU stay	• MV during PICU stay • Vasoactive infusions during PICU stay • Antibiotics during PICU stay • Neuromuscular blockade during PICU stay • Steroids use during PICU stay
8	• Age < 2 years • Post CPR • Circulatory disorder • Myocarditis/cardiomyopathy • PCPC ≥ 2 before admission	• Encephalitis/encephalopathy • Liver dysfunction** • CHD (non-post-operative) • Lung tissue disease (pneumonia) • PIM2 ≥ 1

*Study numbers are consistent with those in Table 4

**Status for non-post operation or post-liver transplantation

*CHD* congenital heart disease, *CPR* cardiopulmonary resuscitation, *ECMO* extracorporeal membrane oxygenation, *FFS* Functional Status Scale, *ISS* Injury Severity Score, *MV* mechanical ventilation, *PCPC* Pediatric Cerebral Performance Category, (*P*)*ICU* (pediatric) intensive care unit, *PIM2* Pediatric Index of Mortality 2, *PRISM* Pediatric Risk of Mortality, *RRT* Renal replacement therapy, *TPN* total parenteral nutrition

We also found that LSPs were significantly younger and were likely to be admitted with an emergency status including post-CPR or circulatory disorders when compared with SSPs. Additionally, LSPs were more likely to have a higher predicted mortality (PIM2). Thus, it is not surprising that these factors were reflected as items in our developed predictive scoring system. In particular, the predictive mortality rate seemed to have significance for LSP prediction, which was assigned 2 points in the scoring system. There may be a reason in the computation method of predictive mortality using PIM2. PIM2 is calculated based on multiple indices such as (1) mechanical ventilation at any time during the first hour in the ICU, (2) emergency admission, (3) admission for recovery from surgery or a procedure, (4) admission following cardiac bypass, and (5) cardiac arrest preceding admission. These indicators have been known to be involved in the patients’ LOS in the ICU [6, 24–26]. Accordingly, PIM2 naturally seems to have been chosen as a significant predictive factor; however, our study showed that our newly developed rule performed better than PIM2 by itself to predict LSPs upon admission. Thus, it could be inferred that our predictive model was a result of correct weighting of PIM2 using additional factors listed on Table 3.

Liver dysfunction for non-operative management, myocarditis/cardiomyopathy, and encephalopathy/encephalitis are the curious indices in our predictive LSP score. We speculate that these factors have significance in LSPs because of their unique treatment modality features.

1. Liver dysfunction for non-operative management
  Acute liver failure in patients who need direct ICU management (i.e., the primary reason for ICU admission was not for post-operative management) could be severe because of fulminant hepatic failure, thus requiring aggressive blood-purification [7]. Moreover, a large number of these patients would require liver transplantation [27], resulting in a longer ICU stay as shown in Additional file 4. There are very few brain-dead donor livers available in Japan; thus, this might contribute to longer waiting times causing prolonged ICU admissions [28].
2. Myocarditis/cardiomyopathy
  It is well known that there is a wide range of disease severities in this disease category. Mechanical circulatory support (MCS) with extracorporeal membrane oxygenation (ECMO) among rapidly deteriorating patients can be imperative for recovery or might act as a bridge to probable heart transplantation. The number of myocarditis patients who undergo MCS has been increasing in Japan [29], placing an additional demand on ICUs for this population. ECMO use during PICU stay has been reported as a factor associated with LSP [5]. A report from a single Japanese center described that myocarditis patients underwent ECMO therapy for median 6 days when ECMO was implemented [30], which is comparative with the findings in this study (Additional file 5).
3. Encephalitis/encephalopathy
  Encephalitis/encephalopathy should influence LOS as a result of its neurological impairment with different ranges as well as the need for treatment using hypothermia therapy [31–33]. Suggested hypothermia protocols require approximately 5 days to complete [32, 33]. One case series from a single Japanese ICU reported that these patients underwent mechanical ventilation for 9.4 days on an average [33]. The use of neuromuscular blockade for temperature control can also influence LOS [5]. The frequent prevalence of encephalitis/encephalopathy in East Asia may involve the statistical significance in our study in Japan [34].

Overall, these unique treatment modalities still need to be validated for their efficacy, despite their practice in some ICUs in Japan. Thus, the management of these diseases would be expected to change due to further research developments.

It is intriguing that our LSP predictive score contains the patients’ status post-liver transplantation. It seems that their LOS depends on their number of ventilation days (Additional file 6). A previous study reported that prolonged mechanical ventilation in pediatric liver transplant recipients had longer ICU LOS, which was associated with younger patient age, preoperative hypocalcemia, and increasing duration of surgery [35]. The addition of this clinical information might improve the specificity of the current LSP predictive score in future studies.

The definition for LOS has been an important discussion point for a long time in the research on LSPs. Previous studies employed different cutoffs ranging from 2 to 30 ICU days. In this study, we attempted to define LSPs using an acceptable approach for everyone [19] and employed a cutoff of 14 ICU days. Eventually, the cutoff point coincided with the maximum number of days that hospitals are allowed to charge for pediatric ICU management according to the 2019 Japanese health insurance system. Thus, our cutoff makes the study more practical for Japanese ICUs.

This study was inherently subject to some other limitations. First, institutional factors, such as the differences in practice, capacity, or patient populations, could not be accounted for in our extracted data. These factors may influence patients’ LOS because the discharge criteria from ICU may vary among facilities. Furthermore, the decision to discharge a patient may not always be based on the criteria; sometimes it is influenced by bed-control matters throughout the hospital or the regional regulation among related hospitals. Thus, the LOS in ICUs may not always precisely indicate the patients’ clinical needs. Second, two variables (sex and chromosomal abnormality) that seemed significant for the prediction of LSPs were unavailable in our validation dataset due to the revision of registration templates during our study period. There might be other factors that were not included in the JaRPAC database that could have predicted a long stay in ICU better. Additionally, the stepwise variable selection we performed in our analysis does not necessarily assure us of the independence of the variables selected in the model. However, as expected, all the remaining variables had ORs of more than one based on descriptive analysis, suggesting limited concerns regarding multicollinearity. Third, the size of our validation dataset was relatively small. Additional verification studies are warranted using external databases or prospectively accumulated datasets in daily ICU settings. Fourth, descriptive patient factors on ICU admission might be statistically important but insufficient to predict the LOS of each patient [5]. Our newly developed LSP predictive score has high sensitivity with low specificity. As LOS prediction aims primarily to improve outcomes for patients and their families, high sensitivity of LSP prediction is required. Conversely, low specificity might hinder the reduction of medical costs and the utilization of resources. Analysis of the association between patients’ outcomes and medical costs or resource consumption in LSPs is warranted in future studies. Fifth, the JaRPAC’s disease categories are registered based on the primary target disease upon admission, which suggest that our analysis might overlook the accompanied conditions (e.g., shock [circulatory disorder] with encephalitis/encephalopathy). Therefore, these viewpoints should be included in future verification studies. Additionally, technological and scientific innovations in ICU treatment could shorten the LOS. Thus, regular revisions would be necessary to accurately reflect the pediatric LSP population.

## Conclusions

Despite being a demographic minority, pediatric LSPs had high rates of bed utilization and mortality in Japanese ICUs. Our newly developed predictive scoring system may help to identify pediatric LSPs upon admission.

## Additional files

Additional file 1: Japanese Registry of Pediatric Acute Care Disease Category. (DOCX 20 kb)

Additional file 2: Distribution of PIM2 against patients’ length of stay (LOS). PIM2 has a negligible correlation with patients’ LOS in the entire study population (*r* = 0.20). Dotted line indicates 15 days of LOS. Each red plot indicates a dead subject. Seven data points are outside the *x*-axis limits. (PPTX 455 kb)

Additional file 3: Clinical outcomes of pediatric patients in Japanese intensive care units admitted with post-out-of-hospital cardiac pulmonary resuscitation status. (DOCX 16 kb)

Additional file 4: Clinical outcomes of pediatric patients in Japanese intensive care units admitted for non-postoperative management of liver dysfunction. (DOCX 17 kb)

Additional file 5: Clinical outcomes of pediatric patients in Japanese intensive care units with the diagnosis of myocarditis/cardiomyopathy. (DOCX 17 kb)

Additional file 6: Clinical outcomes of pediatric patients in Japanese intensive care units primarily admitted for postoperative management of liver transplantation. (DOCX 21 kb)

## Data Availability

The data that support the findings of this study are available from JaRPAC, but restrictions apply to the availability of these data, which were used under permission for the current study, and are thus not publicly available. Data are however available from the authors upon reasonable request and with permission of the steering committee of JaRPAC.

## Abbreviations

AUC: Area under the receiver operating characteristics curve
CPR: Cardiopulmonary resuscitation
ECMO: Extracorporeal membrane oxygenation
ICU: Intensive care unit
JaRPAC: The Japanese Registry of Pediatric Acute Care
LOS: Length of stay
LSP: Long-stay patient
MCS: Mechanical circulatory support
NCCHD: The National Center of Child Health and Development
OHCA: Out-of-hospital cardiac arrest
PCPC: Pediatric Cerebral Performance Category
PIM2: Pediatric Index of Mortality 2
SSP: Short-stay patient

## References

[1] Williams TA, Ho KM, Dobb GJ (2010). Effect of length of stay in intensive care unit on hospital and long-term mortality of critically ill adult patients. Br J Anaesth.

[2] Halpern NA, Pastores SM (2015). Critical care medicine beds, use, occupancy, and costs in the United States: a methodological review. Crit Care Med..

[3] Pollack MM, Wilkinson JD, Glass NL (1987). Long-stay pediatric intensive care unit patients: outcome and resource utilization. Pediatrics..

[4] Namachivayam SP, Alexander J, Slater A (2015). Five-year survival of children with chronic critical illness in Australia and New Zealand. Crit Care Med..

[5] Pollack MM, Holubkov R, Reeder R (2018). PICU length of stay: factors associated with bed utilization and development of a benchmarking model. Pediatr Crit Care Med..

[6] Marcin JP, Slonim AD, Pollack MM, Ruttimann UE (2001). Long-stay patients in the pediatric intensive care unit. Crit Care Med..

[7] Naghib S, van der Starre C, Gischler SJ, Joosten KF, Tibboel D (2010). Mortality in very long-stay pediatric intensive care unit patients and incidence of withdrawal of treatment. Intensive Care Med..

[8] Namachivayam P, Taylor A, Montague T (2012). Long-stay children in intensive care: long-term functional outcome and quality of life from a 20-yr institutional study. Pediatr Crit Care Med..

[9] Brown KL, Ridout DA, Goldman AP, Hoskote A, Penny DJ (2003). Risk factors for long intensive care unit stay after cardiopulmonary bypass in children. Crit Care Med..

[10] van der Heide P, Hassing MB, Gemke RJ (2004). Characteristics and outcome of long-stay patients in a paediatric intensive care unit: a case-control study. Acta Paediatr..

[11] Pagowska-Klimek I, Pychynska-Pokorska M, Krajewski W, Moll JJ (2011). Predictors of long intensive care unit stay following cardiac surgery in children. Eur J Cardiothorac Surg..

[12] Edwards JD, Houtrow AJ, Vasilevskis EE (2012). Chronic conditions among children admitted to U.S. pediatric intensive care units: their prevalence and impact on risk for mortality and prolonged length of stay. Crit Care Med..

[13] Tu JV, Mazer CD (1996). Can clinicians predict ICU length of stay following cardiac surgery?. Can J Anaesth..

[14] Sirio CA, Tajimi K, Taenaka N, Ujike Y, Okamoto K, Katsuya H (2002). A cross-cultural comparison of critical care delivery: Japan and the United States. Chest..

[15] Wunsch H, Angus DC, Harrison DA (2008). Variation in critical care services across North America and Western Europe. Crit Care Med.

[16] Slater A, Shann F, Pearson G (2003). Paediatric index of mortality study G. PIM2: a revised version of the Paediatric Index of Mortality. Intensive Care Med..

[17] Fiser DH (1992). Assessing the outcome of pediatric intensive care. J Pediatr..

[18] Baker SP, O'Neill B, Haddon W, Long WB (1974). The injury severity score: a method for describing patients with multiple injuries and evaluating emergency care. J Trauma..

[19] Weissman C (1997). Analyzing intensive care unit length of stay data: problems and possible solutions. Crit Care Med..

[20] Nupen TL, Argent AC, Morrow B (2016). Characteristics and outcome of long-stay patients in a paediatric intensive care unit in Cape Town, South Africa. S Afr Med J..

[21] Scholefield BR, Gao F, Duncan HP (2015). Observational study of children admitted to United Kingdom and Republic of Ireland Paediatric Intensive Care Units after out-of-hospital cardiac arrest. Resuscitation..

[22] Du Pont-Thibodeau G, Fry M, Kirschen M (2018). Timing and modes of death after pediatric out-of-hospital cardiac arrest resuscitation. Resuscitation..

[23] Makino J, Fujitani S, Twohig B, Krasnica S, Oropello J (2014). End-of-life considerations in the ICU in Japan: ethical and legal perspectives. J Intensive Care..

[24] Ruttimann UE, Patel KM, Pollack MM (1998). Length of stay and efficiency in pediatric intensive care units. J Pediatr..

[25] Ruttimann UE, Pollack MM (1996). Variability in duration of stay in pediatric intensive care units: a multiinstitutional study. J Pediatr..

[26] Kramer AA, Zimmerman JE (2010). A predictive model for the early identification of patients at risk for a prolonged intensive care unit length of stay. BMC Med Inform Decis Mak..

[27] Ide K, Muguruma T, Shinohara M (2015). Continuous veno-venous hemodiafiltration and plasma exchange in infantile acute liver failure. Pediatr Crit Care Med..

[28] Nishimura N, Kasahara M, Ishikura K, Nakagawa S (2017). Current status of pediatric transplantation in Japan. J Intensive Care..

[29] Matsuura H, Ichida F, Saji T (2016). Clinical features of acute and fulminant myocarditis in children- 2nd nationwide survey by Japanese Society of Pediatric Cardiology and Cardiac Surgery. Circ J..

[30] Nosaka N, Muguruma T, Fujiwara T, Enomoto Y, Toida C, Morishima T (2015). Effects of the elective introduction of extracorporeal membrane oxygenation on outcomes in pediatric myocarditis cases. Acute Med Surg..

[31] Kawano G, Iwata O, Iwata S (2011). Determinants of outcomes following acute child encephalopathy and encephalitis: pivotal effect of early and delayed cooling. Arch Dis Child..

[32] Imataka G, Wake K, Yamanouchi H, Ono K, Arisaka O (2014). Brain hypothermia therapy for status epilepticus in childhood. Eur Rev Med Pharmacol Sci..

[33] Nosaka N, Tsukahara K, Knaup E (2017). Intracranial pressure monitoring for pediatric acute encephalopathy. Acta Med Okayama..

[34] Mizuguchi M, Yamanouchi H, Ichiyama T, Shiomi M (2007). Acute encephalopathy associated with influenza and other viral infections. Acta Neurol Scand..

[35] Nafiu OO, Carello K, Lal A (2017). Factors associated with postoperative prolonged mechanical ventilation in pediatric liver transplant recipients. Anesthesiol Res Pract..

