# Supplementary material for: Long-stay pediatric patients in Japanese intensive care units: their significant presence and a newly developed, simple predictive score
Source: J Intensive Care. 2019 Jul 29;7:38. doi: 10.1186/s40560-019-0392-2 (PMC6664501; doi:10.1186/s40560-019-0392-2)
Supplement: Supplementary file 1 — Japanese Registry of Pediatric Acute Care Disease Category. (DOCX 20 kb) [file 40560_2019_392_MOESM1_ESM.docx]

| **Additional file 1. Japanese Registry of Pediatric Acute Care Disease Category** | | |
| --- | --- | --- |
|  |  |  |
| 1^st^ category | 2^nd^ category | 3^rd^ category |
|  |  |  |
| Endogenous | CNS | Encephalitis/Encephalopathy |
|  |  | Seizure/Epilepsy |
|  |  | Cerebrovascular disease |
|  |  | Hydrocephaly |
|  |  | Spinal cord/Vertebra disease |
|  |  | Peripheral nerve disease |
|  |  | Muscular disease |
|  |  | Tumor |
|  |  | Others |
|  | Respiratory system | Upper airway obstruction |
|  |  | Lower airway obstruction |
|  |  | Lung tissue disease |
|  |  | Disordered control of breath |
|  |  | Tumor |
|  | Cardiovascular system | Congenital heart disease |
|  |  | Myocarditis/Cardiomyopathy |
|  |  | Arrythmia |
|  |  | Tumor |
|  |  | Others |
|  | GI system | Hepatoblastoma |
|  |  | Liver disfunction |
|  |  | GI obstruction |
|  |  | GI bleeding |
|  |  | Tumor |
|  |  | Others |
|  | Urorenal system | Renal disfunction |
|  |  | Electrolytes abnormalities |
|  |  | Tumor |
|  |  | Others |
|  | Allergy/Immune system | Anaphylaxis |
|  |  | Others |
|  | Endocrine system | Endocrinopathy |
|  |  | Metabolic disease |
|  |  | Others |
|  | Infection | Sepsis |
|  |  | Soft tissue infection |
|  |  | CNS infection |
|  |  | Respiratory infection |
|  |  | Urinary tract infection |
|  |  | GI infection |
|  | Cancer/Blood system | Hematologic malignancy |
|  |  | Other solid tumor |
|  | Others | Transplant donor |
|  |  | Orthopedic disease |
|  |  | Psychological disease |
|  |  | Others |
|  |  |  |
| Exogenous | Trauma | Multiple injury |
|  |  | Head injury |
|  |  | Others |
|  | Burn | Burn |
|  | Intoxication | Drug intoxication |
|  |  | Others |
|  | Near-drown | Near-drown |
|  | Asphyxia | Asphyxia |
|  | Others | Others |
|  |  |  |
| Unknown | Unknown | Unknown |

CNS, central nervous system; GI, gastrointestinal.
