# Supplementary material for: Long-stay pediatric patients in Japanese intensive care units: their significant presence and a newly developed, simple predictive score
Source: J Intensive Care. 2019 Jul 29;7:38. doi: 10.1186/s40560-019-0392-2 (PMC6664501; doi:10.1186/s40560-019-0392-2)
Supplement: Supplementary file 2 — Distribution of PIM2 against patients’ length of stay (LOS). PIM2 has a negligible correlation with patients’ LOS in the entire study population (r = 0.20). Dotted line indicates 15 days of LOS. Each red plot indicates a dead subject. Seven data points are outside the x-axis limits. (PPTX 455 kb) [file 40560_2019_392_MOESM2_ESM.pptx]

## Slide 1
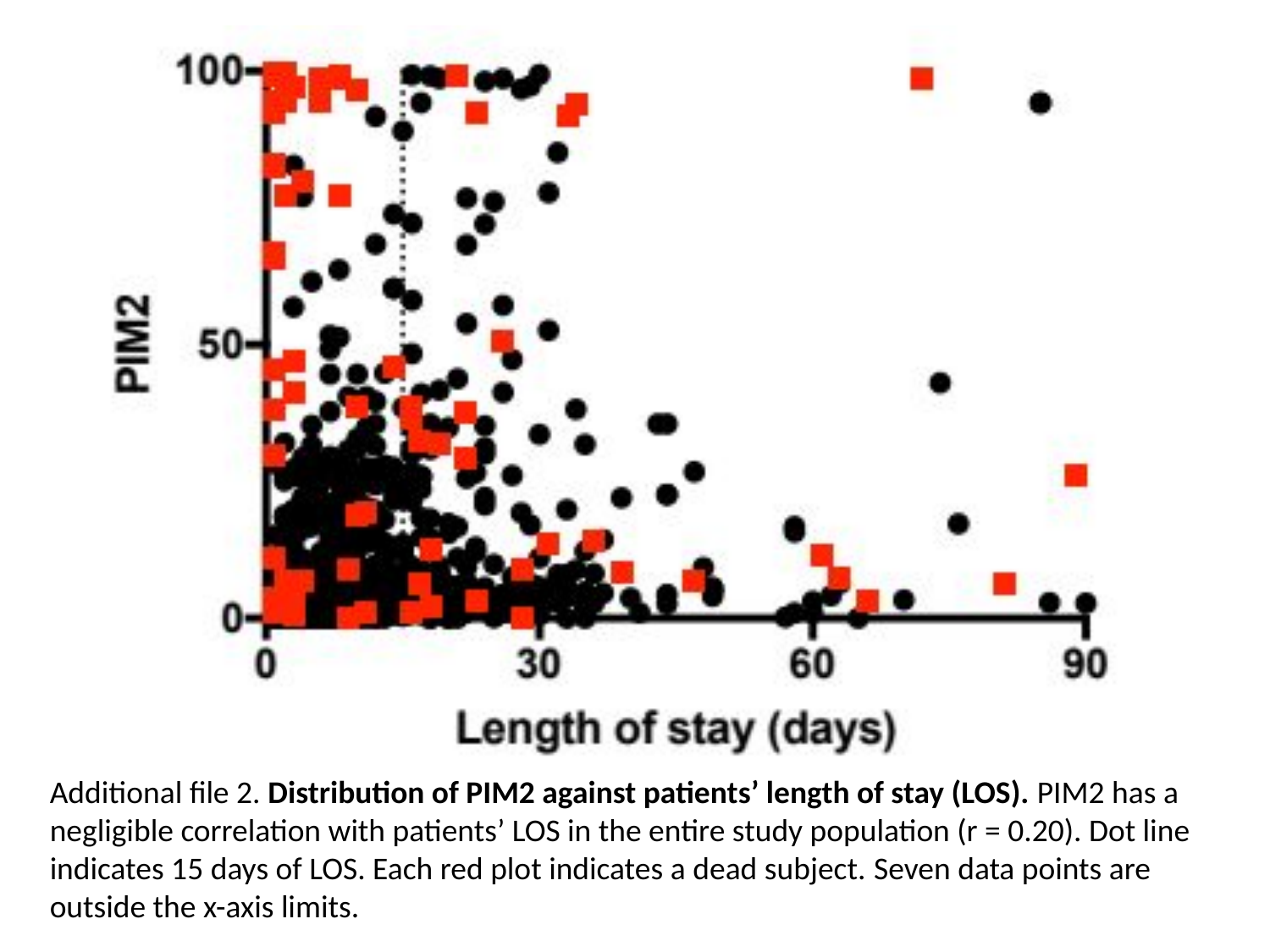

Additional file 2. Distribution of PIM2 against patients’ length of stay (LOS). PIM2 has a negligible correlation with patients’ LOS in the entire study population (r = 0.20). Dot line indicates 15 days of LOS. Each red plot indicates a dead subject. Seven data points are outside the x-axis limits.
