# Supplementary material for: Long-stay pediatric patients in Japanese intensive care units: their significant presence and a newly developed, simple predictive score
Source: J Intensive Care. 2019 Jul 29;7:38. doi: 10.1186/s40560-019-0392-2 (PMC6664501; doi:10.1186/s40560-019-0392-2)
Supplement: Supplementary file 3 — Clinical outcomes of pediatric patients in Japanese intensive care units admitted with post-out-of-hospital cardiac pulmonary resuscitation status. (DOCX 16 kb) [file 40560_2019_392_MOESM3_ESM.docx]

**Additional File 3.** **Clinical outcomes of pediatric patients in Japanese intensive care units admitted with post-out-of-hospital cardiac pulmonary resuscitation status**

|  | All Patients  n=41 | SSPs  n=28 | LSPs  n=13 |
| --- | --- | --- | --- |
| Length of stay (days)  Average (SD)  Median (IQR) | 13.0 (12.0)  9.0 (3.0-21.0) | 6.2 (4.3)  5.5 (2.0-9.8) | 29.0 (8.8)  29.0 (21.0-34.0) |
| Mortality [numbers (%)] | 14 (34.1%) | 12 (42.9%) | 2 (15.4%) |
| PCPC 4 or 5 on discharge [numbers (%)] | 16 (39.0%) | 7 (25.0%) | 9 (69.2%) |

IQR, interquartile range; LSPs, long-stay patients; PCPC, Pediatric Cerebral Performance Category; SD, standard deviation; SSPs, short-stay patients.
