# Supplementary material for: Long-stay pediatric patients in Japanese intensive care units: their significant presence and a newly developed, simple predictive score
Source: J Intensive Care. 2019 Jul 29;7:38. doi: 10.1186/s40560-019-0392-2 (PMC6664501; doi:10.1186/s40560-019-0392-2)
Supplement: Supplementary file 4 — Clinical outcomes of pediatric patients in Japanese intensive care units admitted for non-postoperative management of liver dysfunction. (DOCX 17 kb) [file 40560_2019_392_MOESM4_ESM.docx]

**Additional File 4.** **Clinical outcomes of pediatric patients in Japanese intensive care units admitted for non-postoperative management of liver dysfunction**

|  | All Patients  n=77 | SSPs  n=45 | LSPs  n=32 |
| --- | --- | --- | --- |
| Age (month) median (IQR) | 12.0 (4.0-37.0) | 14.0 (4.0-69.0) | 10.5 (4.0-21.0) |
| PIM2 median (IQR) | 6.8 (3.9-22.9) | 5.3 (1.3-7.5) | 22.5 (5.9-32.1) |
| Length of stay (days) Average (SD)  Median (IQR) | 15.2 (15.9)  11.0 (3.0-18.0) | 5.3 (3.8)  4.0 (2.0-8.0) | 29.1 (16.0)  24.0 (17.0-34.0) |
| LT during ICU stay [number (%)] | 28 (36.4%) | 4 (8.9%) | 24 (75.0%) |
| Live donor [number (% LT)] | 26 (92.9%) | 4 (100%) | 22 (91.7%) |
| Dead donor [number (%LT)] | 2 (7.1%) | 0 | 2 (8.3%) |
| Blood purification [number (%)] | 36 (46.8%) | 10 (22.2%) | 26 (81.3%) |
| Blood purification days Average (SD) | 8.3 (5.6) | 4.8 (2.0) | 9.7 (6.0) |
| Median (IQR) | 7 (5.0-8.5) | 4.5 (3.3-6.5) | 7.5 (6.0-11.0) |

IQR, interquartile range; LSPs, long-stay patients; LT, liver transplant; SD, standard deviation; SSPs, short-stay patients; NPPV, non-invasive positive pressure ventilation.
