# Supplementary material for: Long-stay pediatric patients in Japanese intensive care units: their significant presence and a newly developed, simple predictive score
Source: J Intensive Care. 2019 Jul 29;7:38. doi: 10.1186/s40560-019-0392-2 (PMC6664501; doi:10.1186/s40560-019-0392-2)
Supplement: Supplementary file 5 — Clinical outcomes of pediatric patients in Japanese intensive care units with the diagnosis of myocarditis/cardiomyopathy. (DOCX 17 kb) [file 40560_2019_392_MOESM5_ESM.docx]

**Additional File 5.** **Clinical outcomes of pediatric patients in Japanese intensive care units with the diagnosis of myocarditis/cardiomyopathy**

|  | All Patients  n=52 | SSPs  n=34 | LSPs  n=18 |
| --- | --- | --- | --- |
| Age (month) median (IQR) | 14.5 (7.8-44.0) | 15 (7.0-53.8) | 13.5 (8.3-31.5) |
| PIM2 median (IQR) | 21.9 (4.9-40.8) | 12.5 (2.5-27.5) | 39.6 (30-53.6) |
| post-CPR　　　　　 [numbers (%)] | 5 (9.6%) | 1 (2.9%) | 4 (22.2%) |
| Length of stay (days)  Average (SD)  Median (IQR) | 13.2 (17.6)  8.5 (2.8-21.0) | 5.3 (3.7)  5.0 (2.0-8.0) | 28.1 (23.3)  22.0 (21.0-24.8) |
| ECMO [numbers (%)] | 14 (26.9%) | 5 (14.7%) | 9 (50.0%) |
| ECMO days |  |  |  |
| Average (SD) | 14.2 (27.9) | 5.6 (1.1) | 19 (34.6) |
| Median (IQR) | 7.0 (5.3-8.8) | 6.0 (5.0-6.0) | 8.0 (7.0-10.0) |
| Mortality [numbers (%)] | 3 (5.8%) | 1 (2.9%) | 2 (11.1%) |

IQR, interquartile range; LSPs, long-stay patients; SD, standard deviation; SSPs, short-stay patients.
