# Supplementary material for: Long-stay pediatric patients in Japanese intensive care units: their significant presence and a newly developed, simple predictive score
Source: J Intensive Care. 2019 Jul 29;7:38. doi: 10.1186/s40560-019-0392-2 (PMC6664501; doi:10.1186/s40560-019-0392-2)
Supplement: Supplementary file 6 — Clinical outcomes of pediatric patients in Japanese intensive care units primarily admitted for postoperative management of liver transplantation. (DOCX 21 kb) [file 40560_2019_392_MOESM6_ESM.docx]

**Additional File 6.** **Clinical outcomes of pediatric patients in Japanese intensive care units primarily admitted for postoperative management of liver transplantation**

|  | All Patients  n=97 | SSPs  n=72 | LSPs  n=25 |
| --- | --- | --- | --- |
| Age (month) Median (IQR) | 11.0 (7.0-44.0) | 13.0 (8.0-62.5) | 9.0 (7.0-23.0) |
| PIM2 Median (IQR) | 3.1 (1.1-4.1) | 3.0 (1.1-4.0) | 3.3 (1.2-4.1) |
| Length of stay (days) Average (SD)  Median (IQR) | 13.7 (13.4)  9.0 (7.0-15.0) | 8.0 (2.0)  8.0 (7.0-9.0) | 30 (18.2)  22.0 (17.0-35.0) |
| Live donor [Numbers (%)]  Dead donor [Numbers (%)] | 95 (97.9%)  2 (2.1%) | 71 (98.6%)  1 (1.4%) | 24 (96.0%)  1 (4.0%) |
| Mechanical ventilation [Numbers (%)] | 59 (60.8%) | 39 (54.2%) | 20 (80.0%) |
| Ventilation days Average (SD) | 5.1 (8.4) | 2.3 (0.8) | 10.8 (12.9) |
| Median (IQR) | 2.0 (2.0-3.5) | 2 (2-2) | 7.5 (2.0-14.3) |
| NPPV [Numbers (%)] | 29 (29.9%) | 15 (20.8%) | 14 (56.0%) |
| NPPV days Average (SD) | 8.6 (6.6) | 4.0 (2.0) | 13.4 (6.3) |
| Median (IQR) | 7.0 (3.0-12.0) | 3 (2.5-5.5) | 12.5 (9.3-17.3) |

IQR, interquartile range; LSPs, long-stay patients; NPPV, non-invasive positive pressure ventilation; SD, standard deviation; SSPs, short-stay patients.
